# Morphometric Identification of Queens, Workers and Intermediates in *In Vitro* Reared Honey Bees (*Apis mellifera*)

**DOI:** 10.1371/journal.pone.0123663

**Published:** 2015-04-20

**Authors:** Daiana A. De Souza, Ying Wang, Osman Kaftanoglu, David De Jong, Gro V. Amdam, Lionel S. Gonçalves, Tiago M. Francoy

**Affiliations:** 1 Departamento de Genética, Faculdade de Medicina de Ribeirão Preto, Universidade de São Paulo, Ribeirão Preto, São Paulo, Brazil; 2 School of Life Sciences, Arizona State University, Tempe, Arizona, United States of America; 3 Norwegian University of Life Sciences, Department of Chemistry, Biotechnology and Food Science, Aas, Norway; 4 Departamento de Biologia, Faculdade de Filosofia Ciências e Letras de Ribeirão Preto, Universidade de São Paulo, Ribeirão Preto, São Paulo, Brazil; 5 Universidade Federal do Semi-árido, Mossoró, Rio Grande do Norte, Brazil; 6 Escola de Artes, Ciências e Humanidades, Universidade de São Paulo, São Paulo, São Paulo, Brazil; University of North Carolina, Greensboro, UNITED STATES

## Abstract

*In vitro* rearing is an important and useful tool for honey bee (*Apis mellifera* L.) studies. However, it often results in intercastes between queens and workers, which are normally are not seen in hive-reared bees, except when larvae older than three days are grafted for queen rearing. Morphological classification (queen versus worker or intercastes) of bees produced by this method can be subjective and generally depends on size differences. Here, we propose an alternative method for caste classification of female honey bees reared *in vitro*, based on weight at emergence, ovariole number, spermatheca size and size and shape, and features of the head, mandible and basitarsus. Morphological measurements were made with both traditional morphometric and geometric morphometrics techniques. The classifications were performed by principal component analysis, using naturally developed queens and workers as controls. First, the analysis included all the characters. Subsequently, a new analysis was made without the information about ovariole number and spermatheca size. Geometric morphometrics was less dependent on ovariole number and spermatheca information for caste and intercaste identification. This is useful, since acquiring information concerning these reproductive structures requires time-consuming dissection and they are not accessible when abdomens have been removed for molecular assays or in dried specimens. Additionally, geometric morphometrics divided intercastes into more discrete phenotype subsets. We conclude that morphometric geometrics are superior to traditional morphometrics techniques for identification and classification of honey bee castes and intermediates.

## Introduction

Honey bee (*Apis mellifera*) females arise from fertilized eggs and normally develop into one of two castes, queens or workers, and their differences are a striking feature of highly eusocial bees [1], [2]. The queen is much larger (around 150-300mg), has a developed spermatheca and 200–400 ovarioles, mandibles with a notch, no corbicula, and a rounded head. In contrast, a worker weighs around 50–110 mg, has 2–12 ovarioles, no or only a very reduced vestigial spermatheca, smooth mandibles, a triangular head and corbiculae on the hind legs for transporting pollen [1], [3], [4], [5]. Honey bee queens and workers also differ in their roles in the colony. While queens produce eggs and pheromones essential for the maintenance of social homeostasis, workers perform all of the other colony tasks, including caring for the queen and brood, comb construction, nest maintenance, defense and foraging [5], [6].

Nurse bees determine the fate of sibling female larvae by controlling food quantity and quality, resulting in two distinct phenotypes: queens and workers. However, *in vitro* feeding can open up the full phenotypic space of honey bee development, including intermediate phenotypes, called intercastes [7]. This type of manipulation reveals that queens and workers are the extreme phenotypes of a distribution of individuals with a wide range of morphological characteristics not normally found in nature [8].

How nutrition affects caste differentiation is a fundamental question of developmental biology in social insects. Studies of mechanisms underlying caste differentiation have been performed since the 1930s [9]. More recently, *in vitro* larval rearing was developed and has been widely used for studying honey bee developmental biology, bee pathology, pesticide effects, and for nutritional evaluations [10], [11], [12], [13], [14], [15], [16], [17], [18], [19], [20], [21], [22], [23].

Morphological analysis is one of the most basic assays in taxonomy. Descriptive analysis of size and shape variation is a fundamental tool for organismal biology studies and has improved considerably in the last few years [24]. With the transition from descriptive morphometrics to quantitative morphometrics, morphological identification has become more accurate and reproducible by taking advantage of new computational techniques [25], [26].

Multiple morphological traits in honey bees, such as ovariole number, hind leg structures (corbiculae), mandibles and stinger shape [27], [28], [29] have been used in adult phenotyping to distinguish female caste traits, with the goal of separating queens from workers. A common approach to phenotyping is to give a categorical score (e.g. 0–3) to the morphological trait; based on this score the individual is categorized as a ‘worker-like bee’, ‘queen-like bee’, or ‘intercaste’ [7]. However, this approach is not quantitative and may not objectively represent differences in multivariate phenotypes. Subtle but important shape changes within the character space of intercastes can be lost by using discrete numerical scores.

Morphometry is a quantitative phenotyping method that analyzes the size and shape of morphological traits. Traditional morphometry focuses on lengths, angles, and areas of morphological structures [25]. Ruttner (1983) showed that traditional morphometry is able to distinguish honey bee queens and workers, since they differ in the size of the head, mandible and basitarsus. A disadvantage of traditional morphometry, however, is that structure shape is not included in the analysis. The shape of a morphological structure is multidimensional, and even making numerous linear measurements of a structure is not sufficient to describe it as a whole, especially when changes are subtle, e.g. the notch in the queen mandible and the degree of development of the corbiculae in the intercastes [25].

Geometric morphometrics is a relatively recent approach that provides a description of the shape by using landmark coordinates. This method employs a comprehensive statistical analysis to extract spatial information from morphological structures, making it more quantitative and accurate than traditional morphometrics [30, 31, 32, 33]. We compared traditional morphometrics and geometric morphometrics in an analysis of *in vitro* reared honey bees in an attempt to more precisely categorize honey bee castes and intercastes using naturally reared queens and workers as reference individuals.

## Material and Methods

### Sample collection

Worker larvae were obtained from three USA commercial lines of Italian honey bee colonies at the Honey Bee Research Facility of Arizona State University, Arizona, USA. Open-mated queens were confined to a comb with a queen excluder cage (46 x 24 x 6 cm), according to Peng et al. [34].

Bees were reared *in vitro* based on established protocol [35], in which 0–24 hour old larvae were grafted directly to the food surface in Petri dishes, with *ad libitum* food (53% royal jelly, 6% fructose, 6% glucose, 1% yeast extract and 34% sterile distilled water). Live larvae were transferred daily to new Petri dishes with fresh food [7]. The Petri dishes with larvae were maintained in an incubator at 34°C and 80% RH until the defecation stage, then were transferred to Petri dishes lined with a piece of sterile filter paper, to avoid fungal growth, and maintained in the incubator under the same conditions until emergence.

Larvae from the same combs that were used for *in vitro* rearing were also used for rearing natural queens and workers. Based on established queen rearing protocols [36], one day old larvae were grafted to plastic queen cups and reared in strong queenless colonies until they emerged. The remaining larvae on each comb were allowed to develop into adult workers. Combs with emerging workers and emerging queen cells were placed in an incubator at 34°C and 80% RH to collect the newly emerged individuals. The natural queens and workers came from the same population and were the same ages as the *in vitro*-reared bees, serving as controls for classification.

Newly-emerged bees from both the artificial rearing environment (N = 116) and the natural environment (queens: N = 30, workers: N = 30) were collected. The bees were weighed (wet weight), and the head, mandibles, hind leg basitarsus, spermatheca and ovaries were collected for further analysis. Ovaries that had more than 15–20 ovarioles were prepared for histological estimates of ovariole numbers [37], since it is difficult to count large numbers of ovarioles during dissection. Ovaries with fewer than 15–20 filaments were counted under a microscope. The head, mandible, basitarsus and spermatheca were placed on microscope slides and photographed with a digital camera attached to a stereomicroscope. Magnification (sufficient to nearly fill the microscope field of view) was held constant for each structure.

### Traditional Morphometrics

**Fig 1:**
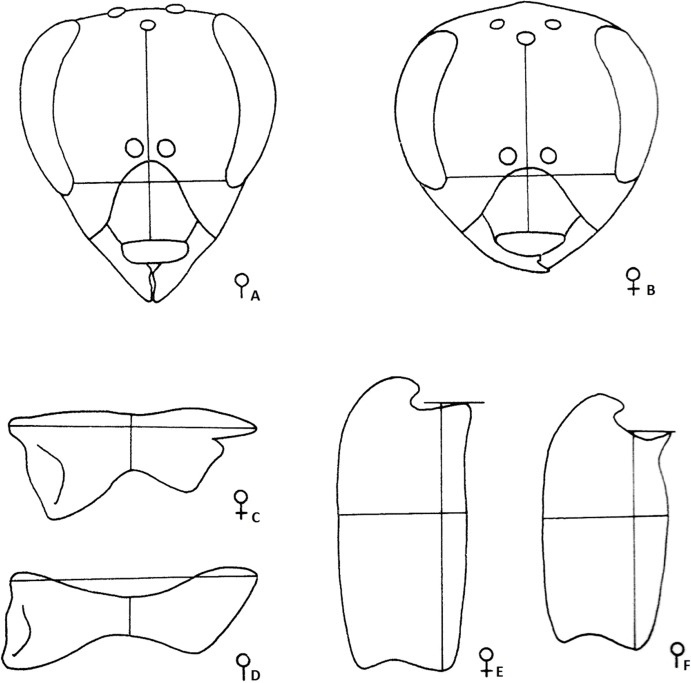
Traditional morphometrics. Diagrams of traditional morphometrics for measurements of morphological structures of both queens and workers: (A) worker head; (B) queen head; (C) queen mandible; (D) worker mandible; (E) queen basitarsus; (F) worker basitarsus. The length and width of each structure were used in traditional morphometrics (Figures modified from Ruttner 1983 [4]).

The traditional morphometrics analyses were made based on a dataset of morphological measures: wet weight at emergence and length and width of head, mandible and basitarsus, as suggested by Ruttner [4] (Fig 1). These measures, along with ovariole number and the size of the spermatheca, form a dataset with nine morphological measures. All measurements were conducted with the aid of the software ImageJ http://rsbweb.nih.gov/ij/.

### Geometric Morphometrics

**Fig 2:**
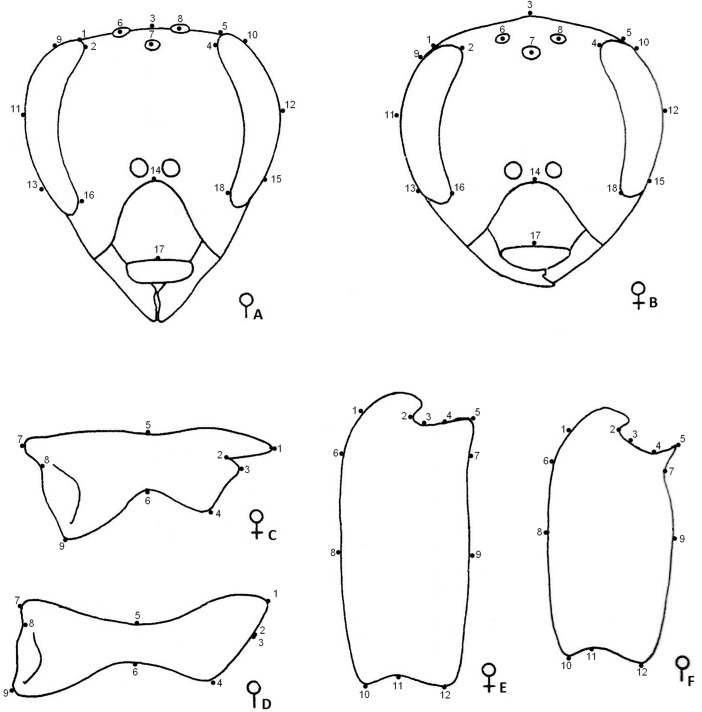
Geometric morphometrics. Diagrams of geometric morphometrics marks of the morphological structures in honey bee queens and workers: (A) worker head; (B) queen head; (C) queen mandible; (D) worker mandible; (E) queen basitarsus; (F) worker basitarsus. Numbers indicate the landmarks that were used in the geometric morphometrics analysis (Figures modified from Ruttner 1983 [4]).

A tps file was made from the images using the software tpsUtil version 1.40 to prepare a databank of the Cartesian coordinates of the plotted landmarks. Landmarks were plotted on the structures using tpsDig2 version 2.12 [38]. We used 18 landmarks for the head analysis, nine landmarks for the mandible and 12 landmarks for the basitarsus (Fig 2). The images were then Procrustes aligned, and the aligned Cartesian coordinates of each landmark were calculated using tpsRelw version 1.45 [39]. Briefly, the images were first scaled to a unit size. After scaling, images were superimposed on the centroids of each structure configuration, and the images were rotated to an optimal fit, exhibiting all the variation in the shape of the structures. The measurements were calculated based on these variations [25]. For more details, see Adams et al. [40].

### Statistical analysis

We used the nine features (weight at emergence, height and width of the head, mandible, and basitarsus, ovariole number and spermatheca size) obtained by traditional morphometrics in a principal component analysis (PCA) in order to classify the *in vitro* reared bees as workers, queens or intercastes, based on distance from the control individuals. For the PCA of the geometric morphometrics, we first performed an analysis with all the information from natural queens and workers, in order to find the main factors responsible for the variability between the two groups with known classification (naturally reared queens and workers). Subsequently, we ran a second analysis using only those variables that contributed most to separating the castes. Both methods were run with and without information about ovariole number and spermatheca size, and compared to each other, in order to determine classification efficiency.

For both morphometric methods we used the Mahalanobis distance from individuals to the centroid of the group to classify each individual. Data were also analyzed with Chi-square tests to compare caste classification made with the two methods. Statistical analyses were carried out using Statistica7 software [41].

## Results

**Fig 3:**
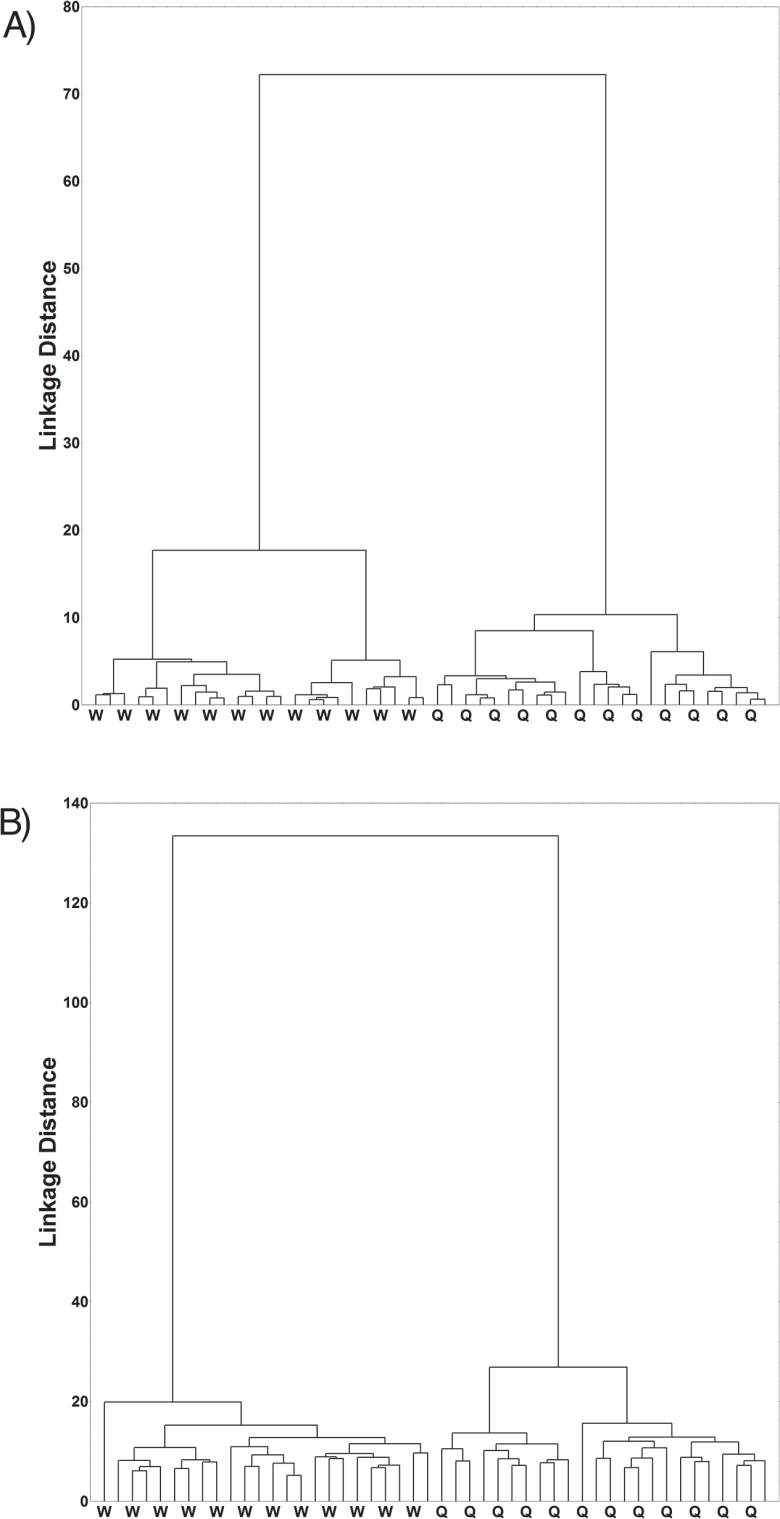
Dendrogram of clustering. A dendrogram of clustering of the naturally developed queens (Q) and workers (W) (*Apis mellifera*): A) Analysis based on traditional morphometric measures and B) Analysis based on geometric morphometric measurements.

Dendrograms were prepared with the data collected by traditional and geometric morphometrics of naturally reared bees. The natural queens and workers were completely separated into two groups with a large linkage distance between them (Fig 3 A and 3 B) with both traditional and geometric morphometrics. However, geometric morphometrics showed greater distances between groups, suggesting that this method is more sensitive to the variation among samples. Then, we added the datasets of *in vitro* reared bees and made further statistical analyses to compare the two morphometric methods.

### Traditional Morphometrics analysis

**Fig 4:**
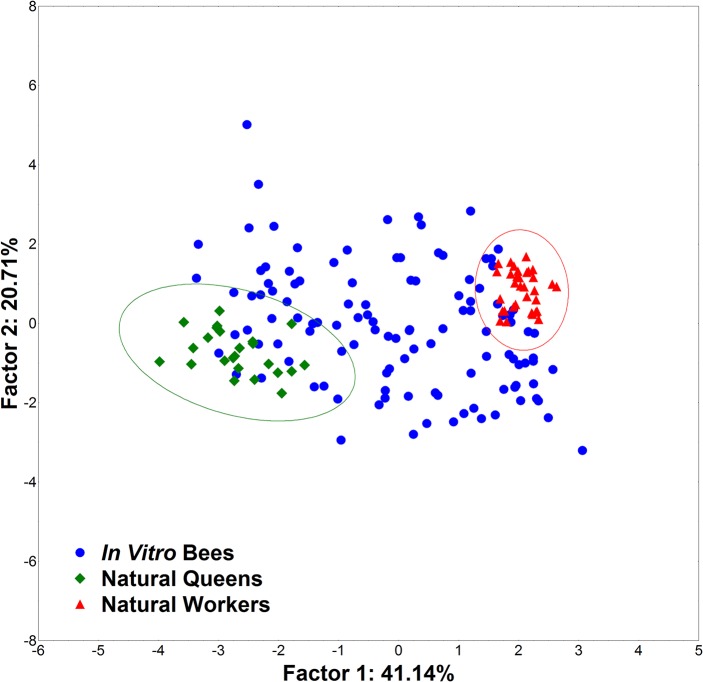
TM1 Principal component analysis. PCA on the data of all morphometric traits produced by traditional morphometrics. Morphological measurements included weight, ovariole number, and sizes of head, mandibles, basitarsus, and spermatheca from *in vitro* and naturally reared honey bees (*Apis mellifera*).

Using the data sets from traditional morphometric measurements, including weight, ovariole number, and spermatheca size, we first established a separation between naturally reared queens and workers. The *in vitro* reared bees occupied the entire space from worker to queen clouds (Fig 4). The Mahalanobis squared distances between the centroids in the PCA of queen and worker phenotypes were used to determine the divergence between queen and worker control groups. Individuals included with 98% confidence in the natural worker and queen ellipses were classified as such, and the intermediates were classified as intercastes. In this first round of analysis, 37 individuals were identified as queens, 33 as intercastes and 46 as workers (Table 1).

**Table 1:** Caste-classification results.

	**TM1**	**TM2**	**GM1**	**GM2**
**Queens**	**37**	**43**	**14**	**14**
**Intercastes**	**33**	**37**	**51**	**53**
**Workers**	**46**	**36**	**51**	**49**

Caste identification in the *in vitro* reared *Apis mellifera*, using both traditional morphometrics and geometric morphometrics. TM1 indicates traditional morphometrics, including all information of weight, ovariole number and sizes of head, mandibles basitarsus, and spermatheca size; TM2 indicates traditional morphometrics, excluding the information concerning ovarioles and spermatheca; GM1 indicates geometric morphometrics, including all information of weight, ovariole number and landmarks of head, mandibles, basitarsus, and spermatheca; GM2 indicates geometric morphometrics, excluding the information on ovariole number and spermatheca).

**Fig 5:**
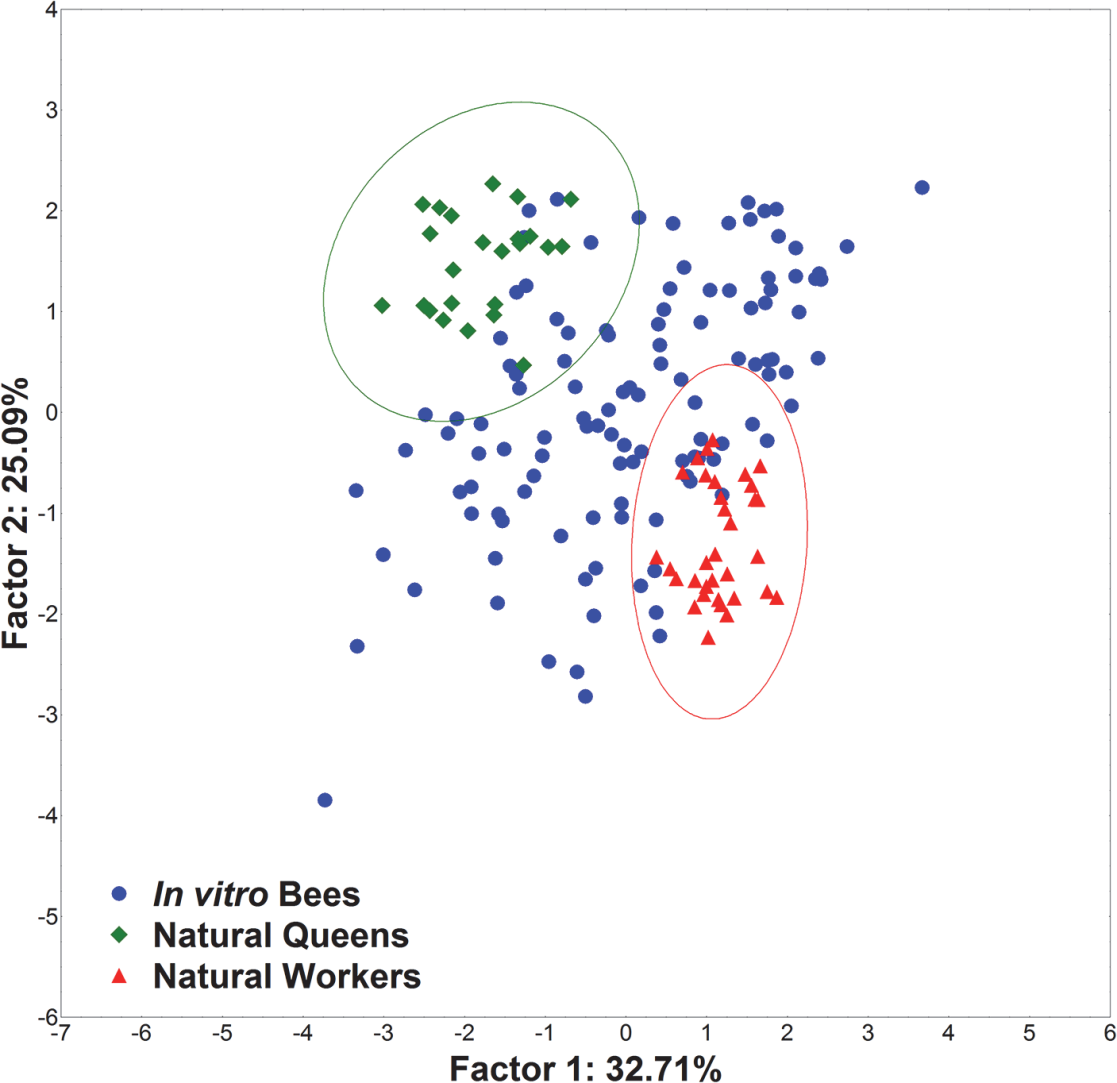
TM2 Principal component analysis. PCA on the data from traditional morphometrics, excluding ovariole number and spermatheca size.

We then performed a second classificatory analysis without the data on ovariole number and spermatheca size, to test the repeatability of the method with reduced information; i.e. only the information concerning weight, head, mandible and basitarsus. We used the same statistical analysis and the same bees as before. With this reduced dataset, we found that 43 *in vitro* reared individuals were classified as queens, 37 as intercastes and 36 as workers (Fig 5).

### Geometric Morphometrics analysis

**Fig 6:**
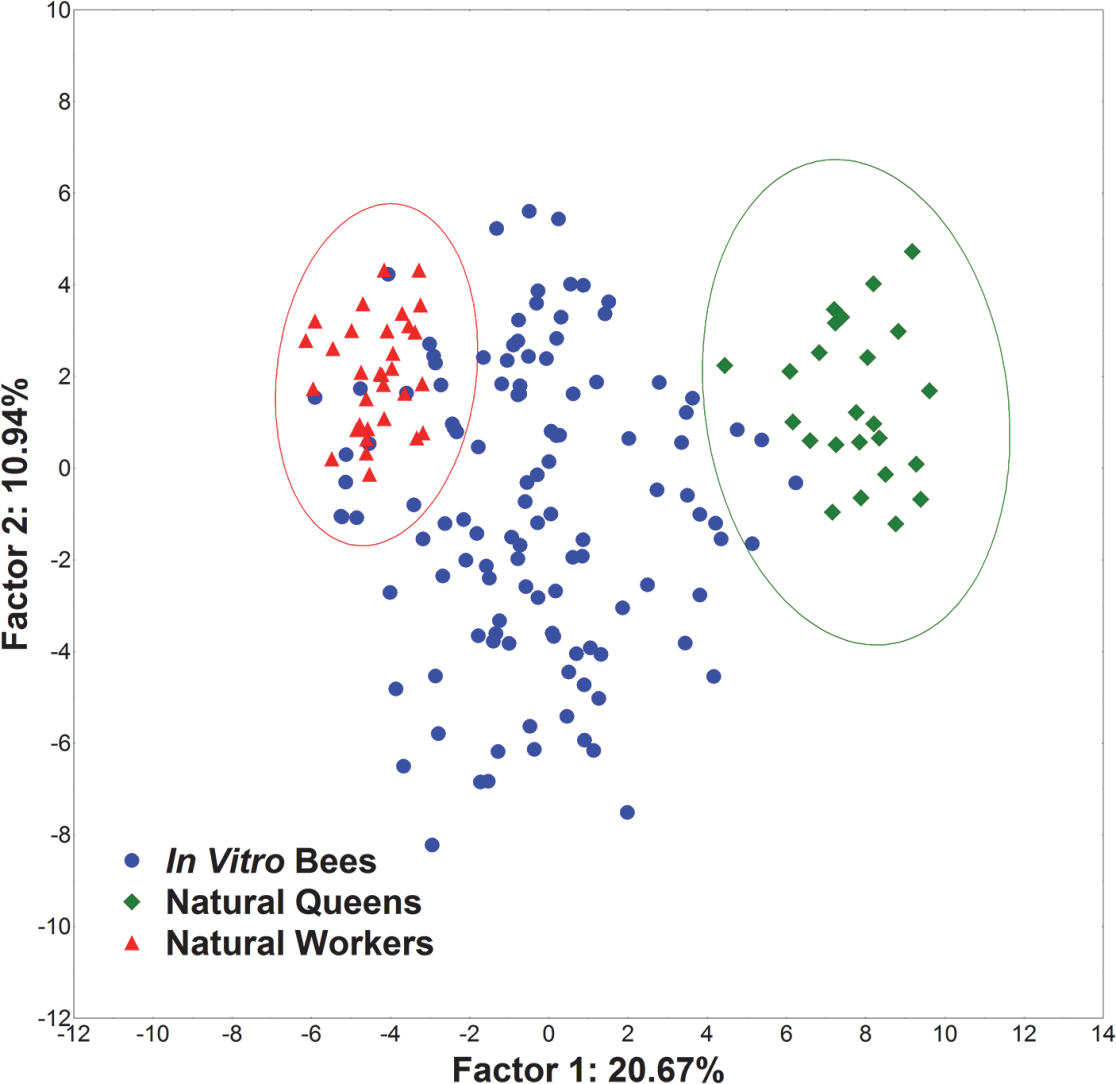
GM1 Principal component analysis. PCA on the data of all morphological traits by geometric morphometrics.

**Fig 7:**
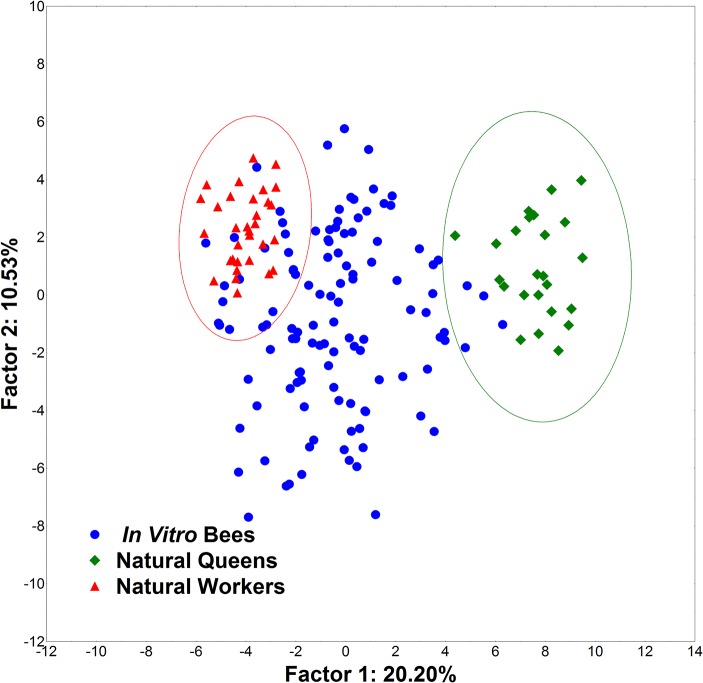
GM2 Principal component analysis. PCA on the traditional morphometrics data, excluding ovariole number and spermatheca size.

Using weight at emergence, ovariole number, spermatheca size and the 39 Cartesian landmarks generated from the three structures (head, mandible and basitarsus), we also found a significant divergence between natural queens and workers. The same traits of *in vitro* bees were then analyzed, and three categories (queen, worker and intercaste) were determined based on the distances from natural queen and worker phenotypes (Fig 6). The Mahalanobis squared distances between the centroids of the queen and worker groups were also significant. In this analysis, 14 individuals of *in vitro* reared bees were classified as queens, 51 as intercastes and 51 as workers (Table 1). Next, we analyzed the same dataset with ovariole number and spermatheca removed from the analysis to test the robustness of the method. We found that 14 individuals of the *in vitro* bees were classified as queens, 53 as intercastes and 49 as workers (Fig 7).

Comparing the classifications derived by traditional morphometrics, with and without the ovariole number and spermatheca information, we observed no significant divergence (Chi-Square = 4.04 df = 2 p < 0.1321); this was also true for the comparison between geometric morphometrics with and without ovariole number and spermatheca information (Chi-Square = 0.15 df = 2 p < 0.9244). When traditional morphometrics, including all characters, were compared with geometric morphometrics, the results were significantly different (Chi-Square = 24.65 df = 2 p < 0.0001); the same was true with the more limited data set (Chi-Square = 31.17 df = 2 p < 0.0001). Compared to traditional morphometrics analysis, the absence of ovaries and spermatheca measures had much less effect on caste classification by geometric morphometrics (Table 1).

## Discussion

The *in vitro* reared bees occupied the entire space between natural worker and queen clouds of points (Fig 4). Naturally reared queens and workers bees were successfully separated by traditional morphometrics methods. However, traditional morphometrics emphasizes the size of morphological traits, the measurements of which are highly correlated, and it only detects relatively large morphometric variations between groups. In contrast, geometric morphometrics uses spacial data, which can be 2D or 3D, allowing it to detect subtle variations in morphological structures.

The traditional morphometrics results were highly dependent on ovariole number and spermatheca size in the classification of intercastes versus workers and queens. Ovariole number and spermatheca size are major determinants for distinguishing queens and workers; consequently one-dimensional data (width and height of the mandibles, basitarsus and head) may not be sufficient for classifying castes in *in vitro* honey bees.

Geometric morphometrics analysis was less dependent on ovariole number and spermatheca data, since the dataset provides a wider range of morphological variation, making this technique more robust and descriptive. Ovarioles and spermathecae can be inaccessible or hard to obtain, since dissection is necessary; additionally, abdominal structures may have been removed for transcriptional analysis or for other types of genetic investigations.

Regardless of the morphometric method that we used, the phenotypic spectrum of *in vitro* reared bees was consistent with what was found in previous studies about the effects of food on caste determination in honey bees [7], [42]. Accordingly, social regulatory networks control the direction of larval development and consequently adult female phenotypes, driven by nurse bee food provision behavior. Our experimental design, which precludes contact with nurse bees, resulted in a varied spectrum of female forms, from queens to workers, including various intermediate forms, considered to be intercastes, supporting these previous findings. Honey bee intercaste phenotypes normally are not found in colonies, unless larval feeding is artificially manipulated, such as by grafting larvae older than three days for queen production, or naturally in emergency queen rearing when the bees are forced to use old female larvae [43], [44]. However, they would be useful for studies of honey bee development, caste differentiation, and tissue and organ developmental biology. Alternative phenotypes have been found to be useful investigational tools; an example is the isolation of a spontaneous mutation in *Drosophila melanogaster* by Calvin Bridges in 1915 [45], [46]. The production of an intermediate form, the mutant bithorax, led to the discovery of homeotic genes, revolutionizing the field of evolutionary biology [45], [46], [47], [48]. In the case of social bees, a more refined categorization of intermediate caste phenotypes (S1 Fig) would help sort individuals into informative subgroups, which would facilitate detailed studies about aspects of physiology, genetics and epigenetics that differ between these castes.

Geometric morphometrics shifted the way morphological studies are performed, using analyses based on landmarks and outlines. It can be useful as a tool for addressing biological questions [26]. Here, we validated the application of geometric morphometric analysis for honey bee caste identification. Although both methodologies proved to be efficient for the identification of honey bee castes, geometric morphometrics was found to be superior, especially in cases of limited data for discriminating morphological traits. Thus, we suggest that geometric morphometrics be used for segregating phenotypes in developmental studies of honey bees, especially for studies that utilize *in vitro* rearing and similar experimental interventions.

## Supporting Information

S1 Fig Dendrogram with the categorization of morphotypes based on morphological phenotypes in comparison to hive-reared bees. Sample identification is magnified (abbreviations, Nat. Q = Natural queen, Nat. W = Natural worker and InV = *in vitro* reared sample). In the branch indicated as "Intermediate Cluster", it is possible to define five sub-classifications (colored boxes) of these intermediate phenotypes, based on linkage distance as a function of different levels of morphological similarity.
(DOCX)
