# Supplementary material for: Morphometric Identification of Queens, Workers and Intermediates in In Vitro Reared Honey Bees (Apis mellifera)
Source: PLoS One. 2015 Apr 20;10(4):e0123663. doi: 10.1371/journal.pone.0123663 (PMC4404332; doi:10.1371/journal.pone.0123663)
Supplement: S1 Fig — Sample identification is magnified (abbreviations, Nat. Q = Natural queen, Nat. W = Natural worker and InV = in vitro reared sample). In the branch indicated as "Intermediate Cluster", it is possible to define five sub-classifications (colored boxes) of these intermediate phenotypes, based on linkage distance as a function of different levels of morphological similarity. (DOCX) [file pone.0123663.s001.docx]

**Supporting information**


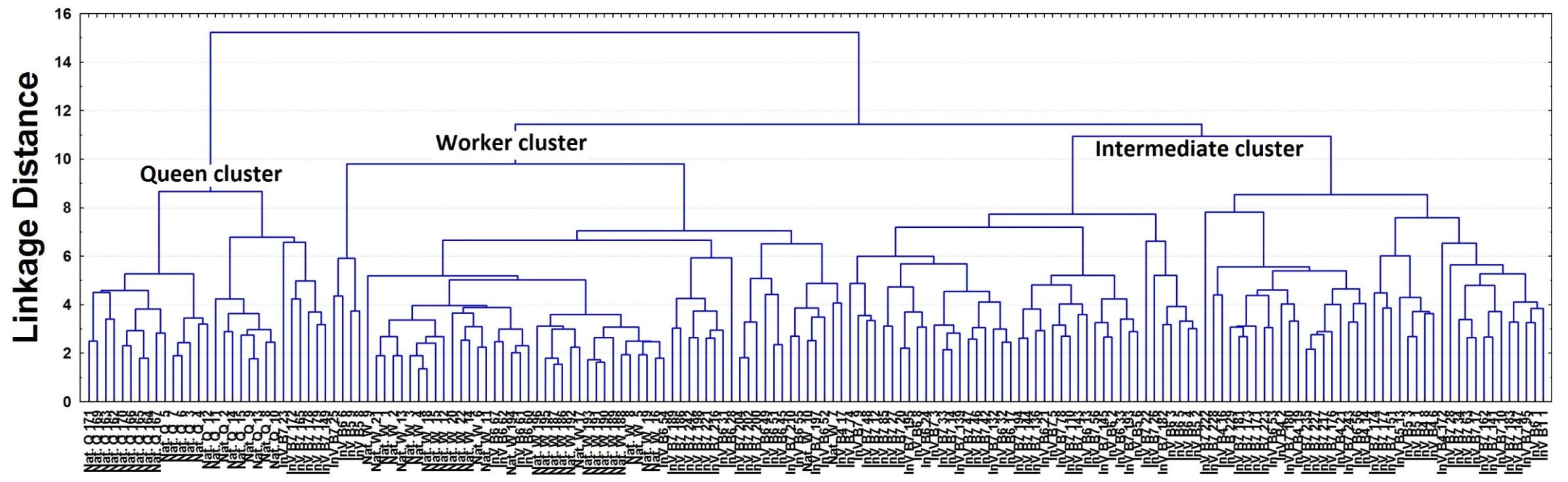

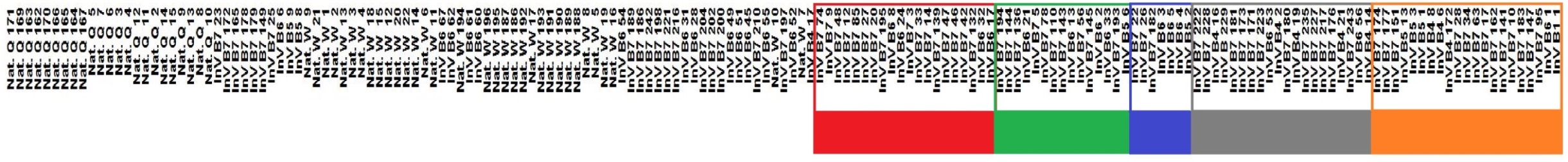


**Figure S1: Dendrogram with the categorization of morphotypes based on morphological phenotypes in comparison to hive-reared bees.** Sample identification is magnified (abbreviations, Nat. Q = Natural queen, Nat. W= Natural worker and InV = *in vitro* reared sample). In the branch indicated as "Intermediate Cluster", it is possible to define five sub-classifications (colored boxes) of these intermediate phenotypes, based on linkage distance as a function of different levels of morphological similarity.
